# Abstract of the Proceedings of the Buffalo Medical Association

**Published:** 1857-10

**Authors:** Austin W. Nichols


ART. II.—*Abstract of the Proceedings of the Buffalo Medical Association.*

TUESDAY EVENING, Sept. 1, 1857.

The association met at the usual hour.

Present—Drs. Rochester, Hamilton, White, Strong, Gay, Eastman, Wilcox, Nichols, Newman, Nichell, Lemon, Treat, A. Flint, Jr., Ring, Hutchins, Boardman, Baker, and Miner.

The President, Vice-president and Secretary, being absent, on motion, Prof. Rochester was appointed president, *pro-tem*, and Dr. A. W. Nichols, secretary, *pro-tem.*

The minutes of the last meeting were read and approved.

*Dr. Treat* then introduced a patient to the society, for diagnosis. A scrofulous-looking girl, aged 15 years, very much emaciated; states, that she had been perfectly well and quite fat up to last Christmas; was then seized with pain in the side, not severe; began to emaciate; had attacks of diarrhœa off and on; did not suffer much from pain; abscesses formed on neck, and discharged a quantity of pus. *Now*, has some febrile movement; there is great emaciation, particularly marked in the extremities; there are now several small abscesses on neck, which are discharging; has a good and even keen appetite; no diarrhœa at present; drinks considerable, and passes a large quantity of water; the abdomen is tumid, a peculiar rigidity of the

---

Amer. Med. Gazette, vol viii, new series, p. 106.

muscles existing. Her aspect is decidedly morbid, presenting the scrofulous cachexia; her mother died of consumption.

(Prof. Rochester having been called away, Prof. White took the chair.).

*Prof. White* having examined the abdomen, thought there was disease of the organs of assimilation, very probably affection of the mesenteric glands.

*Prof. Rochester* stated, that he was struck with the appearance of the patient as she entered the room; he concurred in the opinion of Prof. W., and thought the disease to be that sometimes called *adenosis scrophulosa.*

*Prof. Hamilton* then examined the abdomen of patient; and suggested that the tension of abdomen was not like that due to presence of water, but probably was owing to rigidity of the muscles; he had no doubt that there existed scrofulous disease.

*Dr. Gay* then presented a beautiful specimen of fracture of the neck of the femur with complete bony union. Dr. G. stated, that he was indebted to Dr. Bissell, of this city, for the specimen. The person from whom it was taken was a female, æt. 62 years, residing in this county, the mother of Dr. B—. The fracture occurred from a fall on the hip; Dr. G. was not positive, however, in regard to the nature of the fall. The limb was dressed, splints being applied; these were removed in a short time; and in a few months she regained the use of the limb. Previous to the accident, she had been laboring under an attack of tuberculosis pulmonalis; during the time she was being treated for the fracture, her health improved very much; but she died about four years after she had recovered the use of the limb, from consumption.

*Prof. Hamilton* remarked that the specimen was an interesting one; the capsule was present, (although dry,) which he had not often seen in the specimens of this fracture which he had examined. Though this might be a fracture within the capsule, as was supposed by Dr. Gay, yet it lacked the *positive evidence* of such a fracture. Prof. H. had never seen a specimen of fracture within the capsule with bony union, which has entirely satisfied him. For several reasons, he is led to think that this specimen is not one of fracture within the capsule. First, a fall on the foot would have been much more likely to have produced such a fracture than a fall—as in the present instance—on the hip. Next, although the capsule was attached, yet he was unable to determine that the line of fracture was within the capsule, entirely; he could not see the line of fracture even. Furthermore, the neck of the femur was gone, except about one quarter of an inch above. He could not positively state where the line of fracture was, possibly it might have been across the trocanter major; the direction of the injury would tend to confirm this view. He further stated that the bone was united completely by bone. The shaft was fairly *within* the head of the bone. How did it get there? Not by direct injury. The shaft was sent into the neck, immediately; absorption of the neck then took place, leaving the shaft in the head. He could think of no other explanation. The specimen was a very interestingone; there was complete bony union. Yet, it lacked the positive evidences of fracture within the capsule.

*Discussion on Cent of Nickel and Copper, Coinage of* 1857.

*Dr. A. W. Nichols* reported two cases in which the new cent had been swallowed and passed.

CASE A. Girl, æt. 3 years, swallowed a new cent, coinage of 1857; passed into the stomach with some difficulty, being forced down by successive draughts of water; the parents being alarmed, a dose of ol. ricini was given at night, and the cent passed away the next morning, having been in the alimentary canal about thirty-two hours; no unpleasant symptoms existed during its continuance in the primæ viæ, nor during its exit; the cent was slightly corroded.

CASE B. Girl, æt. 2 years, swallowed a cent, coinage of 1857; passed into the stomach with some difficulty; no medicine was given; it gave rise to no unpleasant symptoms; and passed without inconvenience, in twenty hours after it had been swallowed; this cent was scarcely tarnished.

Dr. N. regretted the absence of Prof. Hadley, in whose possession the last-mentioned cent had been placed. He would state, however, the information communicated to him by Prof. H, in regard to the properties of the constituents of the cent of 1857. This cent was to weigh seventy-two grains twenty-four being copper and forty-eight nickel. Nickel was usually found as an ore, in union with arsecic; but in order to render nickel serviceable for coinage or other use, the arsenic must be entirely driven off or removed. The nickel, therefore, employed in the new coin, was perfectly free from arsenic. Nickel resembles very much the ferruginous metals, and is not injurious when taken into the system. When the new cent is swallowed the danger, if any, arises from the presence of the copper. But as there are but twenty-four grains in this cent, the danger would, of course, be much less than from swallowing one of the old cents. Copper is more readily oxidized and acted on by acids than nickel. But nickel, when alloyed with other metals, as copper, seems to render them less liable to oxidization; this appears to be the case with the new cent.

Dr. N. stated that he had read in the N. Y. papers of cases of poisoning, in children, by swallowing the *new cent;* the physicians having attempted its removal by emetics and cathartics; they noticed symptoms of prostration and of nervous affection which they referred to the poisonous effect of the cent. These symptoms, Dr. N. thought, might be more properly referred to the effect of the remedies employed. As yet, he knew of no instance in which death was stated to have resulted from the ingestion of this coin. He supposed that in most cases no medicine would be required, as the coin itself would serve to increase the peristaltic action of the intestines, and the system would soon rid itself of the foreign body. Emetics would be of no service generally, as the coin lies flat on the mucous membrane of the stomach; and in order for its expulsion, severe contractions of the stomach would have to be produced; if the stomach were full of food, it might be more easily dislodged and removed by an emetic; but the remedy would not be applicable in most cases, on account of its severity.

*Prof. Hamilton* then presented a report of cases of metallic substances taken into the stomach, as follows:

*Nickel and Copper Penny, coinage of* 1857.

Case I. George Richley, æt. 5, swallowed a new cent, Aug. 10, 1857. Forty-eight hours after this his father called upon me for counsel. The child had not yet passed the penny, nor had he experienced any inconvenience from it. The father assured me he would let me know if any ill-effects followed, and as I have not yet heard from him I presume that the lad is well.

*Silver.*

Case II. Ezekiel Annis, æt. 45, swallowed a shilling piece, in 1845. The next day it passed without the aid of physic, and without hving occasioned any inconvenience.

Case III. Willey Begg, æt. 9 months, swallowed a three cent piece in June, 1852. He was then perfectly well. On the following day he had a slight diarrhœa, which continued until the fourth day, when the piece was discovered in the vessel. It was much discolored, but not eroded. After this he took castor oil, and the diarrhœa ceased.

The following example will show that even bogus coin will sometimes "pass."

Case IV. May 24th, 1850, E. S., æt. 3, swallowed a bogus ten cent piece. She took no physic, but on the third day it passed. The evacuation accompanying its discharge was loose, like a diarrhœa; but neither the evacuation preceding nor succeeding were loose. Her health was in no way disturbed while the metal was retained, except as I have mentioned. The piece is discolored, but not eroded.

*Copper.*

Case V. B. M., æt. 7, swallowed a copper penny, and for about half an hour breathed with great difficulty. During the whole of the same day he experienced an uncomfortable sensation in his stomach. No nausea. He took no physic, and his bowels remained regular. On the third day it passed. It was blackened, but not corroded.

Case VI. W. H., æt. 45, swallowed a copper penny. For a day or two he felt a choking sensation; after this he felt a weight in his stomach, which sensation lasted two or three weeks. He never vomited or was made sick in any way. He took no physic. He does not know what has become of it.

Case VII. Ellen M., æt. 7, swallowed a copper cent, Dec. 27, 1849. She was then entirely well. On the following day, and for several days afterward, she vomited everything she eat; and occasionally, when she had not lately eaten, she vomited greenish matter.

March 31, 1849, she was brought to me again. After about a week the vomiting had ceased and she had been well ever since. The cent had not yet passed.

Sometime about the last of April the cent was discovered, having remained in her bowels four months. It was smooth and not in the least eroded, or even discolored.

It is not improbable that in this case the penny was for some days lodged in the lower part of the æsophagus, and that the vomiting was due to this alone. The green fluid was probably only a vitiated gastric secretion.

CASE VIII. Willey Cammen, æt. 4, swallowed a cent, April 1st, 1857. His father soon after brought him to me. He was complaining very much of a pain at the pit of his stomach, which was particularly severe when he attempted to swallow. The attempt was always followed by vomiting. I ordered him a portion of castor oil.

April 6th he was brought to me again. The symptoms remained as when I first saw him. Being persuaded that the penny had lodged above the cardiac orifice of the stomach, I introduced a stomach tube and passed it readily into the stomach, apparently without obstruction. He, however, immediately declared himself relieved, and from this time he experienced no inconvenience. There can be no doubt that the penny was pushed into the stomach at this time. He has remained well ever since; but the parents have not seen the penny.

CASE IX. E. K., æt. 10, swallowed a penny in 1835. Of the result I only know that he is now alive and well.

These are the only cases of swallowing of copper pennies which have come under my own observation, and which I can now call to mind. Some time ago, however, I saw a notice in a newspaper dated June 23, 1851, that a Miss Camberwell, the daughter of a laborer, in England, had "died of mortification of the bowels, by metallic poison, from swallowing a farthing accidentally."

I suspect that the editor was misinformed, and that if she died in consequence of the farthing, it was from mechanical obstruction.

The following is the report of a case found in the 43d vol. of the Boston Med. and Surg. Jour., and copied from the Dublin Quarterly Jour. of Med. Science:

"Dr. Conner detailed to the Cork Med. Society, the history of the following case: A young gentleman, about 18 years of age, called on him in a state of great mental uneasiness, stating that he had, a short time previously, swallowed a copper penny; that he at first made ineffectual efforts to grasp the coin with his finger, and that an apothecary whom he consulted, immediately afterward was equally unsuccessful in his efforts to extract it. A probang was afterward passed without any difficulty, and he was comparatively well for several days, except that his bowels became very costive, so as to require the administration of very active aperients. In about a week from the date of the occurrence, he felt severe pain in the right hypochondrum, about the situation of the pylorus, which became more severe every day, extending up the right side toward the shoulder. Subsequently he had some degree of nausea and vertigo, and complained of a very peculiar distressing sensation, which he discribed as resembling a sudden and violent raising upward of the right side of the body, from the point in which he felt the pain, to the top of the head. This feeling recurred frequently and distressed him very much. When this state had lasted a few days, he suddenly discharged a large quantity of blood by the bowels, and very soon after a quantity of clotted blood by vomiting.

"The ordinary remedies were had recourse to, but the hæmorrhage continued until the patient expired, about four and twenty hours from the first discharge of blood. The apothecary who was in attendance, stated that, immediately after death he distinctly felt the coin in the part where it was suspected to have been impacted, namely, in the pylorus; but an opportunity was denied of testing the correctness of this opinion by a post-mortem examination.

"Dr. Conner considered the novelty of the occurrence of death from such a cause a sufficient reason for bringing the case under the notice of the society, more particularly as in books there is more generally found a recital of the extraordinary substances that have been swallowed and passed through the alimentary canal without producing much injury, than of the exceptional cases in which death has been produced by swallowing objects apparently less calculated to cause danger."

*Gold.*

Case X. Mr. F., of Buffalo, aged about 30 years, swallowed, accidentally, a $20 gold piece. It lodged in the æsophagus. Dr. S., of Buffalo, attempted to remove it, but did not succeed. Subsequently I made the same attempt, and in the second effort I unintentionally pushed it into his stomach. Two years afterward it had occasioned him no inconvenience, nor was he aware that it had ever passed.

*Pins.*

Case XI. *A brass pin.* The infant daughter of A. C. H., of Buffalo, aged 2 years, swallowed a brass pin, June 3, 1849. On the same day the parents called upon me. I ordered no remedies. The pin has never been seen, nor did it ever occasion any inconvenience.

Case XII. July 22d, 1852, Mrs. H., of Buffalo, swallowed a small pin. She felt it distinctly as it was passing down the æsophagus, during several hours. Three days after, when she consulted me, she had experienced no inconvenience. I have not heard from her since.

Case XIII. Miss ——, of Buffalo, swallowed a pin in 1855. She was very much alarmed at the probable consequences, and soon consulted me. I advised her to permit it to take its own way. After two years she tells me that she never has experienced any inconvenience from it, nor does she know what has become of it.

The following remarkable case was mentioned to me some years since by the mother of the child:

Case XIV. *Tenpenny Nail.* Henry Lava, aged 8 months, swallowed, in 1848, a tenpenny nail. The child had at the same time a hernia. In two days it was distinctly felt in the hernial sac, and a surgeon pushed it from the sac back into the belly. In two weeks more it passed the rectum, having never occasioned any inconvenience to the little patient.

In connection with which it may be well to place the case found in the London Med. Chi. Rev., vol. iii, (1825), second ser., p. 301:

Case XV. *Iron Nail.* "A child, 18 months old, at Pembroke Dock, swallowed an iron nail about an inch long, having a rough head. Strong convulsions soon followed, and then spontaneous vomiting. These subsided, and on the fourth day the stools became black and evidently ferruginous, with retention of urine. This last symptom being relieved, the child went on till the seventh day, when vomiting and convulsions again recurred. The same train of symptoms took place once more on the fourteenth day; after which the child suffered no further inconvenience, and perfectly recovered its health. The black stools never appeared but once, and the nail could not be detected in the motions."

Case XVI. *Brass Button.* Edward R., 2 years old, of Buffalo, swallowed a small brass button, Feb. 9, 1853, and passed it on the 2d June, 1853, having retained it about four months. He took no medicine, and suffered no inconvenience from it. It was discolored wnen he swallowed it, but quite bright and polished when it came away.

Case XVII. *Glass Door-knob.* Dr. S. has informed me that when 8 years old, he swallowed a portion of a glass door-knob; the knob being a little broken so that it was irregular in form, some of the angles being quite sharp. Its longest diameter was a little more than one inch, and its shortest three-quarters of an inch; weighing 162 grains. He took castor oil every day, for three days, and kept still. On the third day it passed. He never experienced any inconvenience from it, except that on the first day there was a sensation of weight in his stomach. A few years ago Dr. S. kindly sent the knob to me for examination, and I can confirm the correctness of his statements as to its size, form, &c.

For the following references to examples of metallic or other hard and indigestible substances received into the stomach, this society is indebted to Messrs. Gilbert, Monahan, and Harvey, medical students:

*Bone in Stomach; treated by muriatic acid; recovery.* Lon. Med. Chi. Rev., old series, vol. i, p. 480.

*American Knife Eater; death.* Ibid, vol. iv, p. 218.

*Accidental Knife Swallowing; death.* Ibid, 2d series, vol. ii, p. 476.

*Copenhagen Needle Patient.* Ibid, vol. iii, p. 559.

*Iron, Steel. Pewter, &c., in Stomach of a Lunatic.* Ibid, vol. xxxiv, p. 456.

*Iron Spoon; death.* Edinburgh Med. & Surg. Jour., vol. xxxv, p. 224.

*Piece of a Delft Plate; death.* With remarks, &c. Ibid, vol. xxxvi, p. 56, (1331.)

*Accumulation of Cotton in Stomach; removed by emetics, &c.; recovery.* Buffalo Med. Jour., vol. v, p. 741.

*Thirty-eight pieces of various hard substances, including two Compasses and a Knife. Idiot boy. Death.* Philosophical Trans., Jores abridgement, vol v, p. 278.

*Piece of Stave, nineteen inches long, twenty-two other pieces of wood, &c. Death.* Dictionnaire des Sciences Méd. Art. Cas Rares.

*Knives Extracted.* Ephémérides, Dec. 2, Ann. 10, art. 9, L'Histoire de Prusse, Partie 2.

*Silver Fork Extracted; recovery.* Rapport des Travaux de l'Academie Royale de Méd. de Bordeaux, Ann. 1828.

*Bleeding of Stomach from mechanical causes: such as Pins, Blades of Knives, &c. Resulting in Death.* Acts of the Med. Soc. of Berlin, vol. i. p. 53. Nouvelles de Repub. de Lettres, art. 10. Bonetus, Sepulchretum Anat., Lib. 3, sect. 44.

(*Two cases of Calculus in appendix vermiformis; ulceration; death*. Amer. Jour. Med. Sci., vol. xxxi, p. 122; also vol. xxxii, p. 196.)

*Silver Spoon; excision from intestines.* New York Med. Repository, vol. x, p. 367. 1807.

*Leaden Bar, ten inches long. Gastrotomy. Recovery. Operation by*

*Dr. Bell, of Wapello, Iowa.* Med. Examiner, vol. xi, p. 183. Philadelphia. 1855.

*One pound and nine ounces of Pins. Death and autopsy.* The Med. Exam., vol. x, p. 123. 1854.

*Various articles.* Brodie' Clinical Lectures, p. 172.

See also, Liston's Surg., by Mutter, p. 332. Gibson's Elements of Surg., vol. ii, p. 53. Chelius's System of Surgery, with Notes, by South, vol. iii, p. 102.

Mr. Jansen, also relates that John Hen swallowed a gold breast pin. His brother advised him to eat as much as he could of "pate de pain d'epices," a very tenacious dough. Two days afterward he passed the pin entangled in hardened fæces.

These do not constitute all which a careful and thorough examination of the journals and books would probably bring to light, and I wish that some one who has both the leisure and the inclination, would arrange and analyze them all so as to make them useful for reference and study.

I must not omit to relate to you before I close, one most remarkable case, but which you will not find in the books:

A boy had swallowed a silver dollar. None of the faculty could devise any mode of relief, whereupon the inventor of the Cure-All was sent for. "It is evident," said he, "that so considerable a coin can never be thrown up by any drug known to science, but you shall witness soon the surprising efficacy of my invention." The medicine was administered in some West India treacle, and an hour afterward the boy threw up the dollar, but in small change, principally in five cent pieces.

*Dr. Strong* stated that he was acquainted with an instance of a child who had swallowed an old cent, occurring in his own practice, the circumstances of which were so remarkable, that he would furnish a detailed account of the case, to the secretary, for publication.

*Dr. Boardman* would call attention to the case of Mr. Flersheim, in Prof. Hamilton's report. He had seen Mr. F. two years after he had swallowed the coin; he then had no knowledge of having passed the coin, and had experienced no ill-effects therefrom.

The members of the society expressed their opinion in regard to the swallowing of foreign bodies, more particularly metallic substances, and furnished cases as follows:

*Dr. Eastman*, some time since, saw a child five or six years old, who had swallowed a cent of the old coinage; the penny remained there three days without producing any unpleasant effect. The Dr. desiring to remove the coin, gave mild cathartics, and on the fifth day the cent came away, without inconvenience and having produced no ill effect. The remedies were resorted to, as Dr. E. deemed it prudent to get rid of the foreign body.

*Prof. Hamilton* does not think the old copper penny ever proved injurious, except by producing mechanical obstruction.

*Dr. Miner* was called to see a boy aged three or four years, who had swallowed an old penny; no ill effects were manifested; no medicine was given; cannot say, whether or no it passed away.

*Dr. Newman* related the case of a child, 3 yeers old, who had swallowed a button, a common brass coat-button; there was slight nausea. Dr. N. advised a hearty meal and a dose of oil; button came away in three days, having produced no unpleasant symptom.

*Dr. Treat* knew of a child who had swallowed a piece of lead; advised parents to wait, and it would work its way through, by its own weight; it passed in due time.

In connection with the passage of bodies by their own weight, he related an anecdote which Dr. Musser was in the habit of telling his class: "A man was so situated that he found himself in need of a cathartic, and no medicine was to be had in all the country round. The old lady where he was stopping, becoming acquainted with his necessities, retired and shortly came into the room with a bowl full of water in which there was rolling a bullet. 'What's this?' said he. 'Oh! do n't be afraid of it,' says she, 'it'll cure you; swallow it; *it has been through me three times already.*' "

*Dr. Ring* reported a case of a boy, 6 years of age, who had swallowed a Spanish quarter; had some difficulty in swallowing it; had pain in œsophagus; bread was given, which forced the coin down into the stomach; it produced no injurious symptoms, and came away on the third day, or sixty hours after he had swallowed it.

*Prof. White* referred to the popular impression in regard to the new cent, and the value of the cases reported as tending to dispel it. Prof. W. then reported the case of an infant, ten months old, who swallowed a new cent; no medicine was given; it passed through the following day. Also, another case in which the new cent was not corroded. Still another case in Orleans Co.; there was no corrosion of the new cent; no unpleasant symptom was manifested. Prof. W. further stated, that large bodies pass through the intestines without injury. A child swallowed an old cent; it passed through him in two or three days, without any ill effects. The case of Capt. M., was very remarkable, and he would relate it as told him by the Capt. Having been shipwrecked, and desiring to preserve a *bull's eye watch* which he prized highly, he swallowed it, and in due time it came away. Not wishing to lose it, he swallowed it the second time, and a *second time* it passed through him; both times without pain or inconvenience.

As to the propriety of giving remedies, Prof. W. was of the opinion that emetics should not be given, as there was little likelihood of removing the foreign body by means of them. Cathartics he would not advise, unless constipation existed or was produced.

*Dr. Boardman* stated, that a friend of his, had informed him of the case of a German, whom he saw near Rochester, last July; this man made his living by the money given him for swallowing pebbles. The day previous he had swallowed 172 pebbles; at that time, he swallowed a handful of ordinary sized pebbles. There could be no deception nor sleight of hand, in this instance. The stones passed through him without the aid of remedies.

(The President, Dr. Flint, appeared, and took the chair.)

*Dr. Hutchins* related the case of a gentleman who takes pebbles as food. He reported the case of a man who swallowed a piece of copper about the size of a prune; it came away in two days, without creating any pain in the dejection.

*Prof. White* coincided with Prof. Hamilton in regard to the danger being entirely from mechanical obstruction in the vast majority of cases; and thought that generally no treatment would be required.

*Prof. Rochester* reported a case of a child, 4 years old, who had swallowed a piece of brass, commonly called, a token; no unpleasant symptoms were produced, and in due time it passed away.

*Prof. Hamilton* thought that when an angular body had been swallowed, cathartics would prove injurious; when the body was round, it was not material whether a cathartic were given or no; probably the substance would pass through sooner without one than otherwise.

*Dr. Baker* reported the case of a patient, three or four years old, who had swallowed a new cent; no inconvenience resulted; no medicine was given; he does not know whether it passed or not.

*Prof. White* reported an interesting case in which he employed a novel method of arresting *rectal hæmorrhage.* Patient, male, æt. 26 years, applied to him last winter. Examining him, with Prof. Moore, found a rectal fistula; he had had attacks of hæmorrhage from rectum, but small in amount. An operation was made by cutting from within; the discharge diminished, and the patient improved very much in general appearance; but the cure was not perfect. In June last, patient returned; and Prof. W. discovered another fistula high up in rectum, the lowest point being about 1½ inches from anus, and the highest 2½ inches; it was a sub-mucous fistula. Resolved to operate; at 3, P. M., cut mucous membrane alone, with knife; left patient quite comfortable, having little or no hæmorrhage. At 6, P. M., was called in haste to patient, who had just passed a large quantity of blood; applied cold; soon after, patient had another copious dejection of blood; applied a large roll of cotton to rectum, having enclosed in it a lump of ice. Bleeding continued; syncope came on. There was no external hæmorrhage, but great internal hæmorrhage. Called in neighboring practitioners. Procured a glass tube about an inch in diameter, smooth and open at both ends, which he had been in the habit of using in his obstetrical practice; passed this up the rectum, and making a common *kite-tailed tampon*, filled the rectum as full as possible with it, passing the pieces of lint up through the tube. This arrested the hæmorrhage completely. About twenty-four hours thereafter, removed the the tampon; patient had two large dejections of grumous blood, followed by much relief. In this case, the mucous membrane only was cut, yet the hæmorrhage was severe; it was not rapid, but continuous, and proceeded from many minute vessels.

*Prof. Rochester* reported a case of *perforation of the appendix vermiformis*, followed by peritonitis, and death. Has reported already five cases of this nature, resembling each other very much. This patient was seen in the afternoon; had had slight diarrhœa since morning; was not seen for two days, parents not deeming it necessary, as there appeared to be not much the matter with the patient. When seen next, had general peritonitis; was taken on the 5th of the month, and died on the 8th. Great tenderness existed over the entire abdomen. Prof. R. noticed in this case the same circumstance which he had witnessed in some of the other cases of perforation; during a paroxysm of severe pain, the patient would rise in bed and throw himself violently on the abdomen for relief, like occurs in cases of colic. It is well to observe, that since the decease of this patient, an uncle has had circumscribed peritonitis, but recovered. An aunt had previously died from peritonitis. And the boy himself had had an attack of peritonitis four years before.

*Post-mortem* conducted by Dr. A. W. Nichols, about twenty hours after death. Evidences of general and acute peritonitis were found. Fæcal matter in lower portion of abdominal cavity. No perforation was found except on one side of the appendix near to the terminal extremity. The appendix was quite firmly adherent to the rectum. Being carefully separated and the cecum removed, several small perforations were discovered; this portion of the appendix was enlarged and appeared gangrenous. A hard body occupied its cavity. On laying open the appendix, a hard, oval mass, about the size of a common bean was discovered: this consisted of fæcal matter arranged concentrically around a nucleus of several stony particles of the size of a pin's head.

Prof. R. thinks this case of peritonitis more common than has generally been supposed. He finds the literature of this subject meagre. Rokitansky speaks more fully of disease of the appendix vermiformis than any other writer. He states, that perforation of this portion of the intestines, is not an infrequent occurrence. Besides arrising from obstruction as it does usually, it may occur in the course of typhus fever, tuberculosis, &c.

*Prof. Hamilton* had seen in the medical journals, two cases of gall-stones producing perforation. Was not this case of like nature?

*Prof. Rochester* thought not.

*Dr. A. Flint, Jr.*, then read the following report:

*Case of Poisoning by Syrup of Poppies and Belladonna.*

Case I. The patient was a son of Mr. P., aged two years and six months, He had always been healthy, with the exception of a mild attack of rubeola, from which he was then convalescing. At a quarter to 1, P. M., July 23d, it took, as nearly as could be estimated, an ounce of a mixture of belladonna and syrup of poppies, in the proportion of a grain to the ounce. At half past 1, Dr. Baker saw the patient and administered an emetic of grs. x, zinci sulph., without effect. He then directed some mustard and water, followed by three to four tumblers of tepid water. This also failed to operate. Dr. Lockwood, the family physician, and myself, saw the patient at 3, P. M. Before the arrival of Dr. Lockwood the nature of the poison was not known. We found the patient comatose; prolabia and face much swollen; great difficulty of deglutition, and loss of vision (as far as we could ascertain); on examining the condition of the pupils, we were surprised to find them entirely natural. Another emetic of 10 grs. of sulphate of zinc was immediately administered, but without producing the desired effect. In about thirty-six minutes, 10 grs. of ipecac was given, though it was forced down with great difficulty.

The symptoms gradually increased in severity; the breathing became more labored, and deglutition was now impossible. At about 4 o'clock a full enema of molasses and water was given, and an attempt was made to administer some carb. of ammonia, but the strangling and distress was so great, when any fluid was in the mouth, that it was thought advisable to desist. The breathing was now accompanied by a loud tracheal râle, with frothing at the mouth and nostrils. Pulse very irregular, now soft and slow, now hard and frequent. Still no contraction nor dilitation of the pupils.

Directed sinapisms to the extremities, abdomen, and whole length of the spine. (These were allowed to remain for four or five hours without any apparent effect, though two days afterward the spine was found extensively blistered. All other measures were suspended.

Saw the patient with Drs. Lockwood and Baker, at 6, P. M. The condition was much the same as at the last visit. The same symptoms, certainly not aggravated, and possibly a little moderated. Pulse 160. Directed another enema, as the first had not operated. This operated freely in a few moments.

Saw the patient again at 9, P. M. No perceptible alteration. Still stertor and frothing at the mouth. As it was impossible to administer any remidies, the patient was left to himself for the night.

Saw the patient at ten the next morning, and received the following account: He continued in the same condition in which we left him last night, until about 1 o'clock, when he experienced a change for the better, and continued steadily to improve until we saw him. Dr. Baker called between six and seven in the morning, and Dr. Lockwood at eight, finding the patient very much better. At the time of our visit he was nearly as well as usual. The swelling of the lips and face had entirely disappeared, and his appearance was so much changed that he would not have been recognized. The loss of vision, difficulty of respiration, and deglutition, &c., had entirely disappeared. The pupils were natural, and had not been affected during the entire illness, special attention having been paid to this point.

The above case seems to present points of more than ordinary interest, and I have, therefore, reported it more fully than would at first seem necessary. The great point of interest is the variation of the symptoms both from those of a case of poisoning by belladonna and a case of poisoning by opium.

In Christison's work on poisons, the chapter treating of "compound poisoning, I find a report of the following case:

Case II. "A lady who used a compound infusion of opium and belladonna as a wash for an erruption on the vulva, took it into her head one day to use the wash as an injection; and actually received three successive injections, containing each the active matter of a scruple of opium and half an ounce of belladonna leaves. Fortunately none of the three was retained above a few minutes, except the last, which was not discharged for ten minminutes. In less than an hour, she was found in bed asleep, but the true cause was not suspected till three hours later. She was then completely insensible and motionless, with the face pale, the pupils excessively dilated and contractile; the pulse frequent and small and the breathing hurried. After the use of purgative injections, blood-letting, leeches to the head, and sinapisms to the legs, she began, in five hours, to show some signs of returning consciousness, which improved after a fit of vomiting. When thoroughly roused, her vision continued dim, the pupils excessively dilated, and the ideas somewhat confused. For three days the pulse continued frequent, and the pupils somewhat dilated. Here the opium appears to have prevented the delirium usually induced by belladonna in the early stage, while on the other hand, the belladonna prevented the usual effect of opium on the pupils, and actually produced the opposite action."

Cases of poisoning by belladonna, are not often met with, but I have found reports of two very interesting and distinctive cases: one in the Buffalo Medical Journal for July, 1848, and the other in the American Journal for July, 1857. I shall give a condensed account of the symptoms of these cases, so as to be enabled to compare them with those of the cases just related.

Case III. Buff. Med. Jour. This case occurred at Black Rock, in the practice of Dr. Dayton, and was seen in consultation, by Drs. White and Flint. It was estimated that the patient took two or three ounces of the extract of belladonna, which was prepared by the Shakers, and labelled by mistake, ext. of taraxicum. He shortly complained of vertigo, impaired vision, with dryness of the throat and fauces, and Dr. Dayton was called to see him; he found the pupils very much dilated, and suspected poisoning by belladonna. He administered an emetic of sulphate of zinc and ipecac, which operated satisfactorily; sinapisms were applied to the extremities.

About five hours after taking the poison, Drs. White and Flint saw him. The countenance had become bloated; the mind, which was confused when Dr. Dayton first saw him, had become more affected, the patient replying to questions incoherently and frequently, in a ludicrous manner. There was also great difficulty of deglutition, loss of vision, and disposition to sleep. Carbonate of ammonia was given frequently, and continued throughout the night. Seven hours after taking the poison he could easily be roused, the other symptoms remaining the same. He progressively improved from that time, and by the next morning had nearly recovered.

Case IV. American Journal, July, 1857. This case was recorded by Dr. Wm. Jenner. Poisoning resulted from the continued application of a belladonna plaster to the skin. The symptoms of this case do not differ materially from those of the preceding. There was vertigo, dryness of the throat; loss of vision; great dilitation of the pupils; peculiar delirium; and added to these, a frequent desire to micturate, passing only a few drops of colorless liquid. There is no mention made of bloating of the face.

The treatment was a blister to the back of the neck, an aperient and five grains of carb. of ammonia, every two hours. The patient recovered, and was nearly well on the following morning, though he experienced dimness of vision, and impairment of memory, for several days.

The symptoms of these cases accord very nearly with the description given by Christison in his work on poisons. The delirium is there described as of the pleasing and ludicrous kind as was observed in the cases I have mentioned; dryness of the throat, and difficulty of deglutition are mentioned as pretty constant. In all cases, there is dilitation of the pupil: this is regarded by every one as the most distinctive mark of poisoning by belladonna.

Such cases rarely terminate fatally if they be seen in time to employ the appropriate remedies; when occurring in children, however, they appear to be most serious. Two fatal cases are quoted by Dr. Christison, occurring in children, and terminating within twenty-four hours.

I also find a report in the American Journal, copied from the Lancet, of Aug., 1846, where several persons ate of the belladonna berries, which had been sold as "nettleberries." A man and his wife and two little boys are mentioned. The man, who took them made into a tart, died from the effects of the poison; his wife, who did not eat so heartily, recovered, but labored for some time from its effects. One of the children died on the same day, and the other recovered. No detailed account of the symptoms was given.

The most judicious course of treatment in snch cases, is the following: First it is desirable to evacuate any of the poison which may remain in the stomach, by an emetic of sulphate of zinc, ipecac., salt and mustard, etc., the first being preferable as tts safest and most speedy in its action. The extremities are generally cold, and the patient will usually be benefited by the application of sinapisms. In some cases a blister to the back of the neck or a sinapism along the spine, will relieve a tendency to determination of blood to the head. Christison recommends, in certain cases, the loss of blood from the arm; in this of course we must be regulated by the pulse, etc., as in other cases where bleeding is resorted to. It is desirable, also, to procure an evacuation from the bowels as soon as possible.

The administration of the carb. of ammonia is generally rèsorted to with most happy results; it is recommended by Christison, and we have seen, was administered in cases 2d and 3d, with most gratifying effect. This may be given in doses of five grains every one or two hours, while the patient labors under the effects of the poison. The prognosis is generally favorable.

I have not, of course, attempted to give anything like an elaborate or complete account of the symptoms, treatment, &c., of poisoning by belladonna; but I have merely endeavored to give a sufficiently full account of the two cases which I have quoted, to enable us to make a comparison with cases 1st and 2d.

The quantity of opium estimated to be contained in the amount of syrup of poppies taken by the patient in Case I, is a grain and a half. In a child two years old, this quantity might, and probably would produce, alarming symptoms. Repeated instances are on record of death from five or six drops of tinct. of opium, taken by infants; and such is the peculiar activity of the preparations of opium when administered to young children, that it is always given with unusual care. The quantity of belladonna which was taken in this case, was one grain; this is more than the ordinary dose for an adult, but though a little more than the ordinary dose produces unpleasant symptoms, and though frequently we have them following the administration of a small dose, yet from an examination of the cases reported by Christison and others, we find that a proportional increase of danger is not met with when very large quantities are taken. The fresh berries are most active; and the fatal cases occurring in an adult and a child, mentioned in the American Journal, resulted from taking the poison in this form. In the two fatal cases occurring in children, mentioned by Christison, it was not noted in what form the belladonna was iaken. It seems reasonable to suppose that the danger to the patient in Case I, was mainly due to the opium; while the belladonna impressed peculiarities on the case which made the symptoms nearly resemble those which we see in poisoning by this article. By comparing this case with the cases of poisoning by belladonna alone, we will see many identical features, while two of the most important diagnostic marks of poisoning by belladonna are lost, namely, delirium and dilatation of the pupils. In almost all, if not all cases of poisoning by belladonna, we have delirium in the early stage; this might not have been so marked in a child, but still we would probably have had some approach to it, before stupor set in, instead of an immediate lapse into a state of insensibility, which is generally the case in poisoning by opium. Again, we *always* have dilatation of the pupil in poisoning by belladonna, and this was wanting in case first, the pupils remaining natural during the entire illness. These two points, with an amount of danger to the patient which we could not have had simply from a grain of extract of belladonna, distinctly mark the influence of opium.

The prominent symptom which we had in common with most cases of belladonna poisoning were, swelling of the face, which was noted in Case III, but not in Case IV, great difficulty and finally impossibility of deglutition, which does not exist to so marked an extent in poisoning by opium, and loss of vision.

Case II is the only other case which I have seen, of poisoning by a combination of opium and belladonna: in this case the pupils were very much dilated, and remained so for two or three days, showing that the opposite influences of these changes on the pupil, do not always counteract each other, as in Case I, we would be led to conclude from the special influence on the iris, which belladonna always exerts, whether administered internally or applied to the vicinity of the eye, that in such a case as this, it would be likely to predominate; we can of course form no definite opinion from our present data, having but two cases and those conflicting with regard to this point; but in Case I the opium certainly counteracted the influence of the belladonna on the iris, or vice versa.

Some one has suggested that belladonna be employed as an antidote in cases of poisoning by opium. I am not aware that there was any reason for surmising a favorable effect, excepting the antagonistic influence on the pupils, to which I have just referred. The cases which I have quoted do not certainly show any beneficial influence from the belladonna, but on the contrary, several distressing symptoms not incident to poisoning by opium, were superadded.

*Dr. Lemon* reported the following case:

*Fracture of the right* "*os frontis,*" *with depression; no coma; spontaneous restoration; recovery.*

August 12, 1857, at 9, A. M., Adaline Roach, aged 7 years, fell through the skylight to the ground floor of a four story building on Commercial street, striking on the right frontal eminence. She was taken up pale, and cold, but not unconscious. The depressed portion of bone was in extent the size of a half dollar piece. No external wound other than a contusion; effusion of blood from the left ear; under lip nearly cut through; fracture of the olecronon process of the right ulna. She was very restless; complained of pain in the abdomen, and shortly vomited a great quantity of blood along with the contents of the stomach.

At P. M., reäction had ensued, and she is was in high fever.

Saw her again at eleven; still complains of the abdomen; expresses a desire to void her urine but cannot accomplish it. Considering that she had not urinated since morning early, I introduced a No. 6 catheter and drew off about half a pint of water, upon which she expressed relief. She was never comatose at any period of her illness, but all through it was restless and irritable.

For the first few days the tumefaction of the forehead, nose and eyelids, was very great; it gradually disappeared, however, and the depressed portion of bone rapidly resumed its proper place from natural resiliency, and by the twenty-fifth the depression was scarcely perceptible, and she was entirely well, with the exception of the fractured elbow. On her first attempts to sit up she turned very giddy and faint.

Dr. Hamilton has recorded, and reported, in the Buffalo Medical Journal, for November, 1846, five cases of injuries to the head in children, in which there were two with simply depression; two with fracture and depression, in neither of these did coma supervene; the fifth, however, had persistent coma, after fracture and depression. All made a good recovery.

Whilst on this subject I would inquire what amount of importance is generally attached to the effusion of blood from the ear, after injuries to the skull? Is it a pathognomonic sign of fracture of the base of the cranium?

*Prof. Hamilton* remarked that hæmorrhage from the ear was considered by surgeons, generally, of the present day, as not positively indicative of a fracture; that the subject had always excited a good deal of discussion. He mentioned an instance of a person who fell on a spike, which penetrated the skull, but recovery took place. He had recently seen a case of a child whose skull was fractured, and in which he had to trephine; the child recovered, and in *ten days* after the operation was running about in the streets.

*Dr. Hutchins* related a case of a person who received an injury on the head, in which there was hæmorrhage from the ears, nose, and mouth, with dizziness. This patient recovered, and left the city in six or seven days. Was there fracture at the base of the brain in this instance?

*Prof. Hamilton* did not think these sympioms necessarily indicated such a fracture.

Prof. H. remarked, that every year he was more deeply impressed with the truth, that persons in extremity even, frequently recovered. In this connection he would call to mind the following case:

Was called to see a person in Black Rock, who had fractured the parietal bone, and was in a comatose condition. Prof. H. trephined, although he entertained no hope for the patient. Found dura mater elevated by blood; opened the membrane and blood escaped *per saltum* for some time. Regarding case as hopeless, he left; about half pint of blood having been thrown out. Sent for next morning to visit patient; found him much better; bleeding had continued *per saltum* for some time after he left; but the person began to manifest some signs of consciousness, and had been improving up to time of morning visit. This patient eventually recovered.

There being no further business, the association adjourned.

AUSTIN W. NICHOLS,
Secretary *pro tem.*

